# Growth regulation mechanism of *Rhododendron moulmainense* to high-temperature stress: integrated physiological, transcriptomic, and metabolomic insights

**DOI:** 10.3389/fpls.2025.1680853

**Published:** 2025-10-07

**Authors:** Zaid Khan, Sijia Liu, Jingen Peng, Hongyue Cai, Yuqing Bai, Bing Hu, Luwen Zhang, Peng Zhuang, Zipeng Cai, Lijuan Xie

**Affiliations:** ^1^ College of Architectural Engineering, Shenzhen Polytechnic University, Shenzhen, China; ^2^ Administrative Office of Wutong Mountain National Park, Shenzhen, China

**Keywords:** high-temperature stress, chlorophyll fluorescence, rubisco, starch-sucrose metabolism, ABC transporters

## Abstract

Rhododendrons domestication in urban habitats is limited due to their vulnerability to higher air temperatures, and their adaptive mechanisms are poorly understood. In this study, we evaluated the growth response and regulatory mechanisms of *Rhododendron moulmainense* to high-temperature stress of T35 (35°C) and T42 (42°C). The findings demonstrated that high-temperature stress decreased chlorophyll fluorescence and contents, which was validated by the damaged chloroplast structure in transmission electron microscopy. Scanning electron microscopy resulted in reduced leaf stomatal traits, altering gas exchange, and thus, photosynthetic rates were decreased. High-temperature or heat stress (HT) increased the activities of antioxidants and osmolytes under T42 to counteract the damage of reactive oxygen species (ROS). Transcriptome and metabolome analysis using Kyoto Encyclopedia of Genes and Genomes (KEGG) enrichment upregulated 9 differentially expressed genes (DEGs) out of 16 and 9 differentially accumulated metabolites (DAMs) out of 10 of the starch-sucrose metabolism pathway and 11 DEGs out of 16 and 9 DAMs out of 13 of ABC transporters metabolism under high-temperature stress of 42°C to reveal the synergistic effects of these pathways. HT induced expression of genes and metabolites in the starch-sucrose metabolism pathway, which likely increased the photosynthesis and sugar metabolism enzymes such as Rubisco, citrate synthase, sucrose synthase, and sucrose phosphate synthetase. The findings revealed that DEGs and DAMs regulating secondary metabolites (SM) in starch-sucrose metabolism, encoding *SUS, TPS1, BAM1*, sucrose, D-ribose, and D-fructose, and ABC transporters such as *ABCC1, ABCC2, ABCG2*, Thiamine, and Betaine were significantly higher in T42 to regulate the plant growth under HT. These results imply that the interconnected pathways of starch-sucrose metabolism and ABC transporters may help us better understand the growth regulation and domestication processes of *Rhododendron* under high-temperature stress scenarios.

## Graphical Abstract

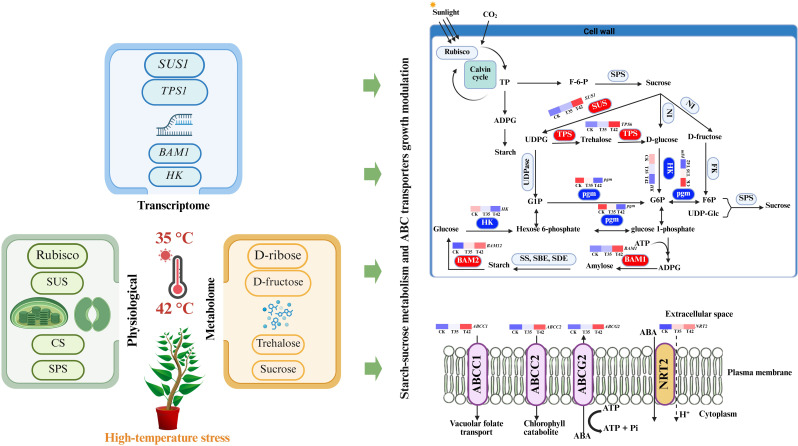

## 1 Introduction

*Rhododendron* species are members of the Ericaceae family, a broad group known for its aesthetic value, medicinal uses, and ecological significance (Liu et al., 2022; Lin et al., 2022). This genus is highly economic and plays a crucial role in the regulation of biodiversity, stability, and sustainability of an ecosystem, particularly forest ecosystems (Zhang et al., 2021). Even though China is home to over half of the world’s *Rhododendron* species (Ma et al., 2014), *Rhododendron moulmainense* cannot be grown at lower elevations because of the increasing temperatures due to climate change, which inhibits its growth and domestication. One of the main factors limiting the use of *Rhododendron* in urban gardening is heat stress, which can impair the fluidity of cell membranes or directly damages them, which inhibits plant growth and development (Luo et al., 2021). The effects of heat stress or other factors on *Rhododendron* growth have all been studied in the past (Lin et al., 2022; Li et al., 2023), but the regulatory pathways that govern the plant’s performance under these environmental conditions remain unknown. Thus, knowing how *Rhododendron* responds to heat stress is critical for maintaining the stability of alpine forest ecosystems and conserving genetic resources.

The rhododendrons are heat-sensitive and cannot withstand excessive cold or heat due to hereditary characteristics, preferring a cool, well-ventilated, and semi-shaded habitat (Li et al., 2020). The introduction and domestication of *Rhododendron* at low elevations are severely hindered by heat stress (Wang et al., 2021). Heat stress causes the overproduction of reactive oxygen species (ROS) in plant cells, which leads to oxidative stress and subsequent cell damage (Duc et al., 2018). Additionally, temperature has a significant impact on plant growth by influencing the demand for carbohydrates required for development and biological activities (Gent and Seginer, 2012). High temperatures can primarily affect the growth and developmental processes of the plants*;* however, the growth regulatory mechanisms of *Rhododendron* under heat stress are still unknown.

Plant secondary metabolites (SMs) are crucial for plant growth and reducing abiotic stressors such as light, drought, salinity, and temperature (Jan et al., 2021). At the molecular level, transcription factors of different genes and metabolites control the accumulation of SMs in response to stress. Plant growth and development are primarily regulated by starch and sucrose metabolism, which play a pivotal role in thermotolerance under high temperatures (Ruan, 2014). Starch and sucrose metabolism initiate the synthesis of ATP and NADPH, which are necessary for nitrogen utilization and ABC transporter activity (Zeeman et al., 2010). The byproducts of starch-sucrose metabolism interact with other signaling pathways to modulate the plant’s performance under abiotic stresses (Chen et al., 2023). In addition to serving as osmolytes to stabilize cellular structures, starch byproducts also empower mitochondrial respiration to sustain ATP synthesis (Chen et al., 2023; Kaplan and Guy, 2004). Moreover, starch-derived sugars induce heat shock proteins that safeguard the photosynthetic apparatus (Larkindale and Vierling, 2008), and sucrose-ABA crosstalk controls stomatal conductivity and water utilization efficiency, respectively (Taji et al., 2002). Glucose is linked to the IAA and the CK signaling pathways, which control the growth, division, and proliferation of plant cells, both synergistically and antagonistically (Zeeman et al., 2010; Taji et al., 2002).

In plants, the accumulation of sugar is dependent on two mechanisms: genes involved in sugar transport and genes involved in sugar production (Wang et al., 2016). ABC transporters facilitate the supply of sucrose, amino acids, and signaling chemicals like abscisic acid (ABA), which control stress mechanisms and the distribution of nutrients like nitrogen (Wang et al., 2016; Kuromori et al., 2010). ABC transporters facilitate the supply of secondary metabolites like flavonoids and antioxidants to reduce the oxidative damage induced by heat stress (Petrussa et al., 2013). A variety of transporters, including ABA transporters, regulate guard cells’ mobility, which modulates plant heat tolerance by decreasing water loss (Jarzyniak and Jasiński, 2014). ABC transporters have been linked to the movement of secondary metabolites that are important for plant defense, pigmentation, and stress tolerance, including flavonoids, alkaloids, and terpenoids (Lefèvre and Boutry, 2018). The intricate regulatory networks that guarantee plant homeostasis and survival are highlighted by the frequent interactions between these secondary metabolites and key metabolic pathways, such as ABC transporters and starch-sucrose metabolism (Yazaki, 2006). The metabolism of starch and sucrose yields ATP and NADPH, which are crucial for nitrogen usage and ABC transporter activities (Zeeman et al., 2010). Thus, the assessment of the interconnected pathways of ABC transporters will further investigate the *Rhododendron* growth-regulating mechanism through sugar accumulation under heat stress.

In order to fill in the knowledge gaps regarding the mechanisms underlying the *R. moulmainense*’s thermotolerance adaptation to high temperatures, the effects of various high temperatures were evaluated using photosynthetic traits, the antioxidant defense system, and transcriptome analysis. We hypothesize that high-temperature stress would decline the plant’s chlorophyll synthesis, photosynthesis, stomatal traits, and leaf cell permeability to affect the growth and developmental processes. We evaluated transcriptome profiles to identify key genes that regulate growth pathways, particularly nitrogen metabolism, ABC transporters, and starch-sucrose metabolism. We hypothesized that intraspecific coordination among these pathways could promote *Rhododendron* growth under heat stress. These integrated and interconnected comprehensive physiological, biochemical, and molecular findings shed light on the growth regulation mechanisms governing high-temperature stress at lower altitudes and present novel insights that will help in future conservation and domestication of *R. moulmainense*.

## 2 Materials and methods

### 2.1 Plant materials and experimental conditions

The response of *R. moulmainense* to heat stress was assessed in October 2024 at Shenzhen Polytechnic University’s College of Architectural Engineering using uniform and robust plants cultivated for three years at the Wutong Mountain Scenic Area Nursery. For seven days, plants were subjected to heat treatments at 35°C and 42°C in a controlled setting with a constant 60% humidity, and a 16/8 light/dark cycle. A temperature of 35°C implies a mild heat stress over the ideal photosynthetic range, which is particularly relevant to Shenzhen’s usual summer conditions. The 42°C treatment was chosen to assess the maximum threshold of plant thermotolerance and simulate a severe heatwave event that seriously damages photosynthetic components. The plants were divided into three groups such as control (CK), heat stress at 35°C (T35), and heat stress at 42°C (T42) for seven days. After seven days, three biological replicates of the leaf samples taken from three separate plants were collected in liquid nitrogen and kept at -80°C for further examinations.

### 2.2 Measurement of chlorophyll fluorescence, chlorophyll contents, photosynthetic rates, and enzyme activities

Chlorophyll content was measured by centrifuging 0.2 g of fresh leaf extract in 80% acetone for 10 minutes at 4°C. After the supernatant was collected, the absorbance for chlorophyll a (chl-a) and chlorophyll b (chl-b) was measured at 663 and 645 nm using a multi-detection microplate reader, CYTATION3, manufactured by BioTek USA. To determine the values of chl-a, chl-b, carotenoid, and total chlorophyll, the methods of (Pei et al., 2010) were used. Utilizing fully expanded leaves of *R. moulmainense*, the LI-6800XT photosynthesis system from Li-Cor Biosciences (Lincoln, Nebraska, USA) fitted with a leaf chamber fluorometer (Li-Cor Part No.6800-40, enclosed leaf area: 2 cm^2^) was used to quantify the fluorescence of chlorophyll. The light source was a combination of blue (10%) and red (90%) LEDs, and the observations were made at a leaf temperature of about 22°C. A portable photosynthesis system (Li-6800, Li-COR Inc.) was used for the measurements of photosynthesis (A), transpiration (E), stomatal conductance (gs), and intercellular CO_2_ (CO_2_ ci) on the fully expanded leaf during the day, from 10:00 am to 3:00 pm, in full sunlight, CO_2_, 400 µmol mol^−1^; leaf temperature, 23°C; light intensity, 1000 µmol m^−2^ s^−1^; and air humidity, 70%. The commercially available kits (Yancheng Mackey Biomedical Testing Service Center Co., Ltd., China) were used to measure the activities of Rubisco, citrate synthase (CS), sucrose synthase (SS), and sucrose phosphate synthetase (SPS). The photosynthesis and sugar metabolism enzyme values were measured at 450 nm of absorbance, and the RubisCO, CS, SS, and SPS values were represented as U/g FW.

### 2.3 Scanning electron microscopy, transmission electron microscopy, and leaf anatomy

Three replicates of a consistent area (1 mm^2^) from the middle sections of the leaves were obtained for each treatment that was chosen. Before being analyzed using electron microscopy, the samples were cleaned with distilled water. To fix the obtained samples, a solution of 4% glutaraldehyde and 0.2 M sodium phosphate buffer (pH 6.8) was employed (6 h, 4°C). This was followed by four rounds of washing with 0.1 M sodium phosphate buffer (pH 6.8). The samples were then cleaned with diluted ethanol, rinsed twice with isoamyl acetate, and then frozen-dried. Double-sided tape was used to secure the leaf fragments to stubs, and gold was used to sputter coat the samples (Kong et al., 2013). A JEOLJSM-6390LV Scanning Electron Microscope was used to examine the samples. Samples were dehydrated using a graded ethanol series and dried using critical point drying before being post-fixed in 1% osmic acid in 0.2 M phosphate buffer (pH 6.8) for transmission electron microscopy. The samples were then examined using a Hitachi 500 electron microscope after the thin leaf pieces were stained with lead citrate and 2% uranyl acetate (Kong et al., 2013).

Leaf samples of 5×5 mm were preserved in FAA solution at 4°C for the whole night. Following that, the samples were washed with 100% propylene oxide and exposed to a sequence of ethanol gradients ranging from 50% to 100%. After that, the samples were immersed in Spurr’s epoxy resin to guarantee fixation. A Leica EM UC7 ultramicrotome (Wetzlar, Germany) was used to cut the leaves into semi-thin sections that were 1 µM thick.

In accordance with the procedure of Zhang et al. (2007), these slices were stained using a 0.01% toluidine blue/sodium borate solution. After removing the surplus staining solution, an AxioCam ERc 5 s digital camera was used to record and view the leaf’s anatomical structure. Lastly, the thickness of the lower epidermis, upper epidermis, palisade parenchyma, spongy parenchyma, and leaf was measured using Image J software (NIH, USA).

### 2.4 Determination of reactive oxygen species and antioxidants

Three biological replicates were obtained from *R. moulmainense* leaves by using pooled samples from three distinct plants that were either under control or heat stress at 35°C and 42°C for seven days after treatment (DAT). The concentration of H_2_O_2_ in the sampled leaves was examined using the methods in the Solarbio kit (BC3950, Beijing, China). Following the guidelines provided by Solarbio (BC0020), the accumulation of MDA in the *R. moulmainense* leaves was measured. In order to determine electrolyte leakage, 0.2 g of fresh leaves were added to 10 mL of distilled water and then placed in a shaker incubator set at 32°C for two hours. After calculating each sample’s EC1 using an EC meter, the samples were heated again at 121°C for 20 minutes and cooled down at room temperature to determine the EC2. The electrolyte leakage was measured using the following formula:

*EL* (%) = *EC*1/*EC*2 × 100, whereas EC1 refers to the initial electrical conductivity and EC2 refers to the final electrical conductivity.

According to the manufacturer’s protocols provided by Solarbio, the relevant assay kits were used to evaluate the enzymatic antioxidant activities of SOD (BC0170), POD (BC0090), and CAT (BC0205) as well as the activities of osmolytes such as ascorbate peroxidase (APX) (BC0225), soluble sugar (BC0030) and proline (BC0295).

### 2.5 Extraction of RNA and library preparation

Following the manufacturer’s instructions, the total RNA was extracted from *R. moulmainense* tissues using the TRIzol reagent (Transgene, ET121-01, Beijing, China). In order to construct the library and Illumina sequencing for transcriptome sequencing, six biological replicates were collected and used by the Illumina Novaseq6000 platform with assistance from Shanghai Majorbio Bio-pharm Biotechnology (Shanghai, China). sRNA library preparation was performed using the Illumina TruSeq Small RNA Kit. The pooled sRNA libraries by (Shanghai Major-bio Bio-pharm Biotechnology) were then sequenced using the Illumina HiSeq2500 platform, producing double-end reads within 18–32 nt. The FASTP software (https://github.com/OpenGene/fastp) was used to filter the raw data, and all raw mRNA-seq reads that qualified the FastQC quality control procedures were trimmed to obtain clean data (reads). The Hisat2 software (http://ccb.jhu.edu/software/hisat2/index.shtml) was then used to map the reads to the genome of *Rhododendron ovatum* (Kim et al., 2015). Only readings that mapped to distinct locations were then subjected to further analysis. To find significant DEGs (fold-change ≥ 2 and *P*_adjust_ < 0.05), gene expression levels were normalized using the EdgeR program. The Gene Ontology (GO) enrichment analyses were conducted using agriGO v2.0 (https://github.com/tanghaibao/GOatools) (Tian et al., 2017). Each gene set was compared to the full genome of *R. ovatum* (Wang et al., 2021) as a background, and GO keywords were deemed substantially enriched with a FDR < 0.05. An online program called omicshare (https://www.omicshare.com/tools/) was utilized for DEGs and KEGG pathway enrichment analysis to perform functional enrichment analysis of differential genes.

### 2.6 Metabolome analysis

After vacuum-freezing the leaf samples in a Scienz-100 F lyophilizer, they were ground into a powder using a Retsch MM 400 mixing mill (30 Hz, 1.5 min). After dissolving 0.1 g of powder in 1.2 mL of 70% methanol extract, the mixture was vortexed six times, once every 30 minutes. Following an overnight storage period at 4°C, the samples were centrifuged for 10 minutes at 12,000 rpm the following day, and the supernatant was then collected, filtered using a microporous filter membrane, and placed in a sampling vial for metabolites analysis. To ensure repeatability, a quality control sample was added to each of the three test analysis samples during the instrumental analysis procedure. The previously reported protocol was followed for the acquisition conditions of chromatography mass spectrometry and the qualitative and quantitative analysis of metabolites (Wang et al., 2019). PLS-DA was used to evaluate the correlations between samples and screen for differential hormone metabolites based on a *P* value <0.05 and a fold change ≥2.0. OmicShare (http://www.omicshare.com/tools) and OmicStudio (https://www.omicstudio.cn/tool) were used to conduct KEGG analysis.

### 2.7 Statistical analysis

The physiological and biochemical characteristics of three replicates of each treatment were examined using statistix 8.1 with one-way analysis of variance (ANOVA) in order to detect significant differences between the different treatments (*p* < 0.05). After ANOVA, the LSD test was used to compare the individual means of the various treatments. OriginPro 2025 was used to analyze the correlation results, alluvial analysis and create the figures.

## 3 Results

### 3.1 Heat stress-induced chlorophyll machinery, photosynthetic traits, and carbon enzymes

**Figure 1:**
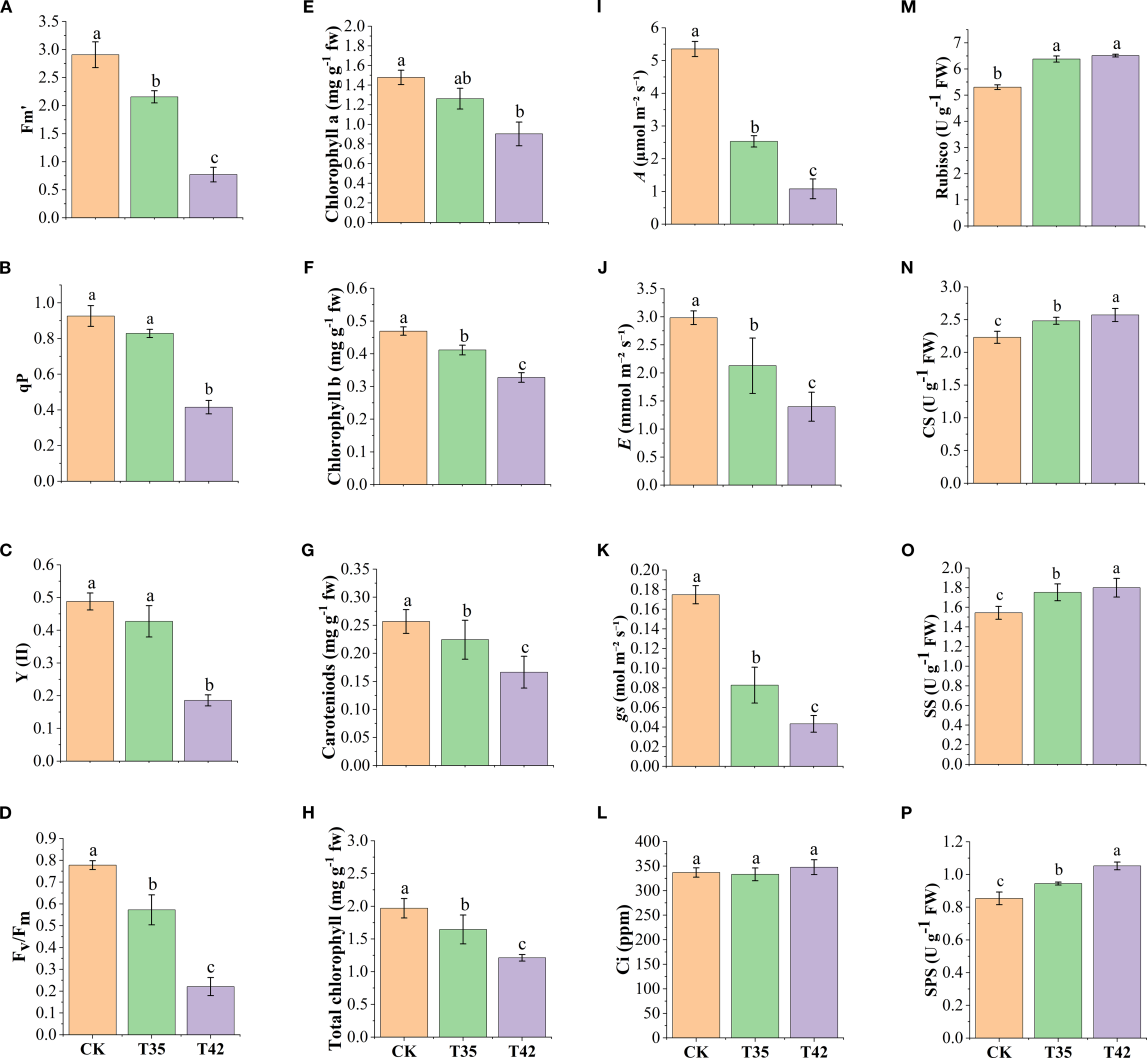
Evaluation of physiological traits of *R. moulmainense* under high-temperature stress. **(A–D)** represent chlorophyll fluorescence, **(E, F, H)** represent chlorophyll contents, **(G)** represents chlorophyll pigments, **(I–L)** represent photosynthetic traits, and **(M–P)** represent sugar metabolism enzymes. Error bars show the standard error for each treatment across three replicates, while various alphabetic letters illustrate the significant differences as determined by the LSD test (*p <* 0.05). CK, T35: 35°C; and T42: 42°C.

To investigate the photosynthetic efficiency of *R. moulmainense* against heat stress, we measured chlorophyll fluorescence, chlorophyll contents, and photosynthetic processes after seven days of high-temperature stress treatment. The findings indicated that the concentrations of maximum fluorescence (Fm), photochemical quenching (qp), the quantum yield of Photosystem II (PSII) (Y (II)), and the efficiency of PSII (Fv/Fm) significantly altered under the treatments of heat stress at 35°C (T35) and 42°C (T42) compared to control (CK) (**Figures 1A–D**). Exertion of heat stress at T42 significantly reduced the levels of Fm, qp, Y(II), and Fv/Fm by 77%, 55%, 61%, and 71%, respectively, as compared to CK (**Figures 1A–D**). The chlorophyll contents, including chl-a, chl-b, total chlorophyll, and chlorophyll pigments such as carotenoids, also decreased in the same trend as chlorophyll fluorescence under the heat stress treatments compared to CK (**Figures 1E–H**). Comparatively, the T42 effect was more adverse than that of T35 and decreased chl-a by 38%, chl-b by 30%, carotenoids by 35%, and total chlorophyll by 38% compared to CK (**Figures 1E–H**).

Furthermore, the photosynthetic rates of *A*, *E*, and *gs* were significantly reduced under heat stress treatments compared to CK; however, the rate of CO_2_ ci remained insignificant (**Figures 1I–L**). The findings revealed that heat stress at T42 reduced the rate of *A* by 79%, *E* by 53%, and *gs* by 75% while increasing the rate of CO_2_ ci by 3% compared to CK. In the current study, we found that when *R. moulaminense* plants were exposed to heat stress, their enzyme activities of carbon metabolism increased. After seven days of heat stress, the activities of Rubisco, CS, SS, and SPS were increased by 22%, 15%, 16%, and 23%, respectively, in response to exogenous heat stress at 42°C compared to CK (**Figures 1M–P**). Likewise, the heat stress at 35°C also recorded increments of 20%, 11%, 13%, and 10%, respectively, in the contents of Rubisco, CS, SS, and SPS. Compared to T35, the effect of heat stress on carbon enzymatic activities was higher at T42 (**Figures 1M–P**).

### 3.2 Heat stress diminished *R. moulmainense*’s leaf anatomical, stomatal, and cell ultrastructural traits

**Figure 2:**
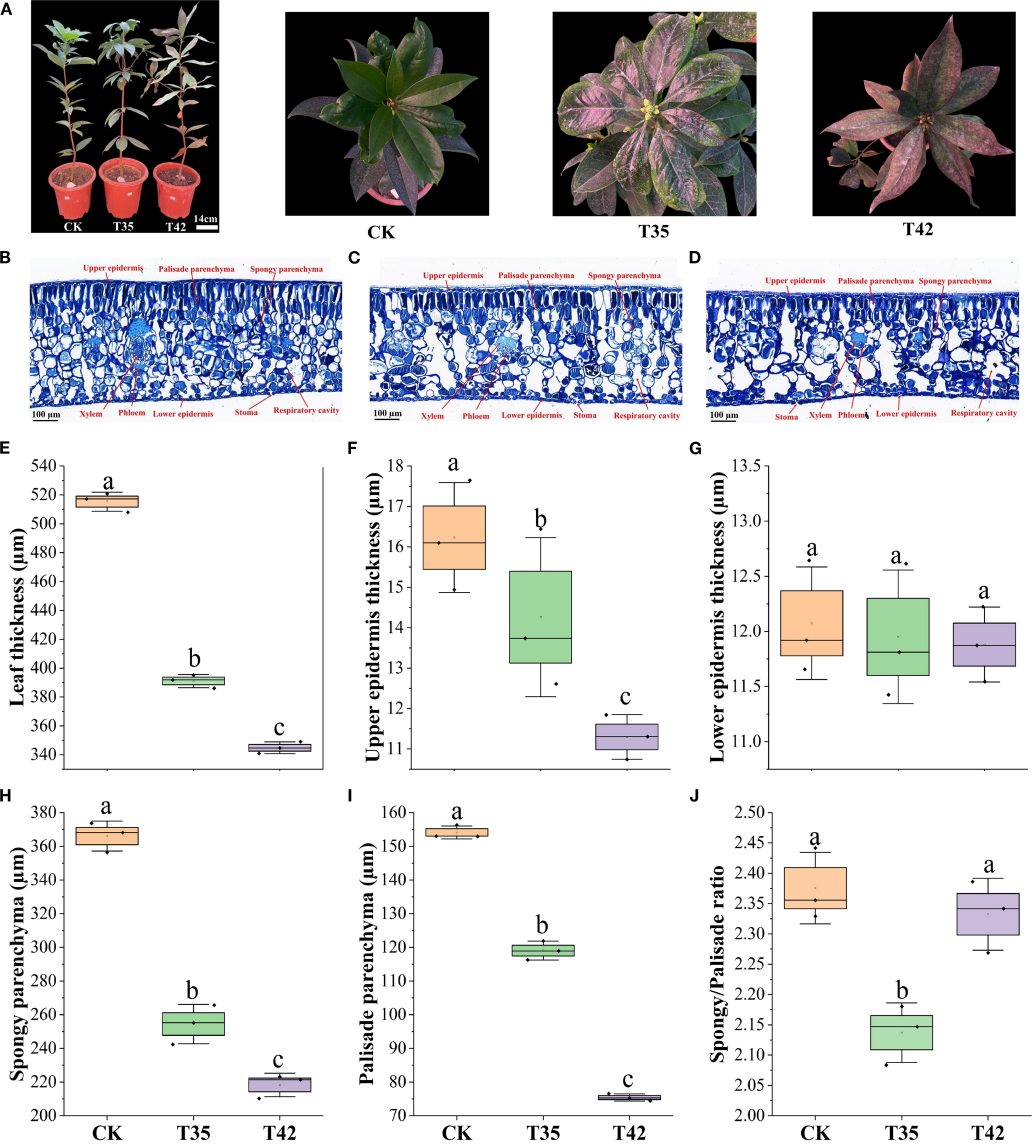
Effect of high-temperature stress on leaf anatomy of *R. moulmainense*. **(A–J)** The figure represents the impact of different treatments (CK, T35: 35°C, and T42: 42°C) on **(A)** phenotypes of *R. moulmainense* representing the treatments. Bar = 100 µm; **(B–D)** The leaf anatomical structures; **(E–J)** The leaf anatomical traits induced by different treatments. Error bars show the standard error for each treatment across three replicates, while various alphabetic letters illustrate the significant differences as determined by the LSD test (*p <* 0.05).

**Figure 3:**
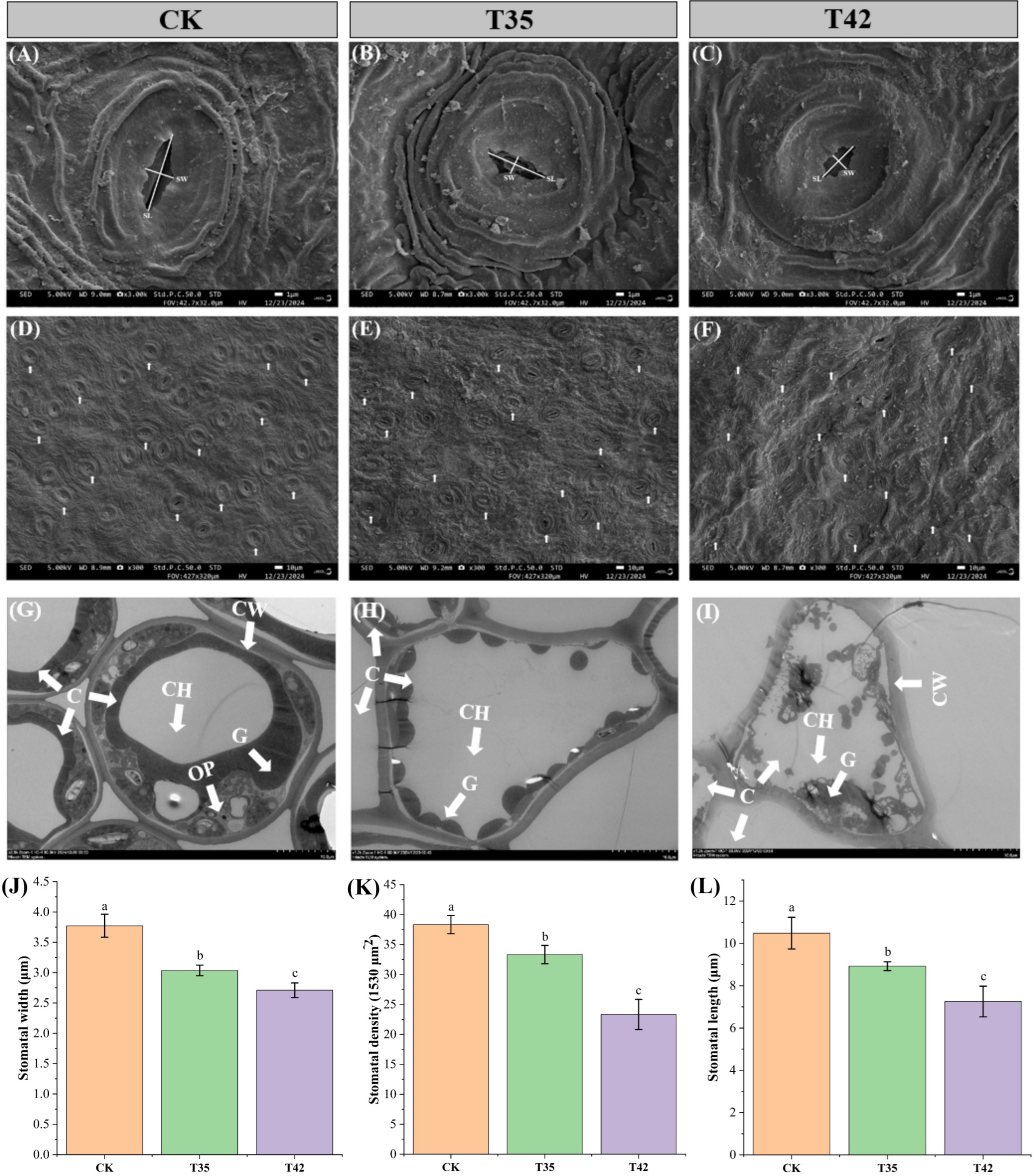
Impact of high-temperature stress on leaf stomata of *R. moulmainense*. **(A–C)** The effect of moderate and severe heat stress on leaf stomatal size; **(D–F)** The effect of moderate and severe heat stress on leaf stomatal density; **(G–I)** The leaf cell ultrastructure under moderate and severe heat stress; **(J–L)** The leaf stomatal traits affected by moderate and severe heat stress. CH, chlorophyll; C, cell; CW, cell wall; and G, grana. Error bars show the standard error for each treatment across three replicates, while various alphabetic letters illustrate the significant differences as determined by the LSD test (*p <* 0.05). CK; T35: 35°C; and T42: 42°C.

Under various environmental conditions, plant growth and development are significantly influenced by cell elongation and cell division. We analyzed the internal structures of the cell anatomy, leaf stomata, and cell ultrastructure to evaluate the effect of heat stress on the growth of *R. moulmainense*, which is evident in the phenotypes (**Figure 2A**). Heat stress at 42°C substantially affected the leaf’s anatomical structure and decreased the thickness of the leaf by 33%, the upper epidermis by 30%, the lower epidermis by 1%, the palisade parenchyma by 51%, the spongy parenchyma by 40%, and the palisade/spongy ratio 1% compared to the CK (**Figures 2B–J**). SEM analysis of *R. moulmainense* leaf revealed that heat stress reduced the stomatal length, width, and density in comparison to the control treatment (**Figures 3A–F**). The leaf ultrastructure analysis from TEM showed that heat stress treatments disrupted the shape and shrank the size of the cell, unlike the cells in the control treatment, which had improved starch grains and a better cell wall structure with fine and transparent edges (**Figures 3G–I**). However, the cell wall thickness was increased by 90% in T42 treatment as compared to CK (**Supplementary Figure S2**), to protect structural integrity, reduce water loss, and prevent cells from oxidative and thermal damage. The results showed that the heat stress of T42 significantly reduced the stomatal length by 30%, stomatal width by 28%, and stomatal density by 39% compared to CK treatment (**Figures 3J–L**). Comparatively, the heat stress at T42 was very adverse to damage the leaf stomatal, anatomical, and ultrastructural traits as compared to T35.

### 3.3 Heat stress elevated the activities of reactive oxygen species, osmolytes, and enzymatic antioxidants

**Figure 4:**
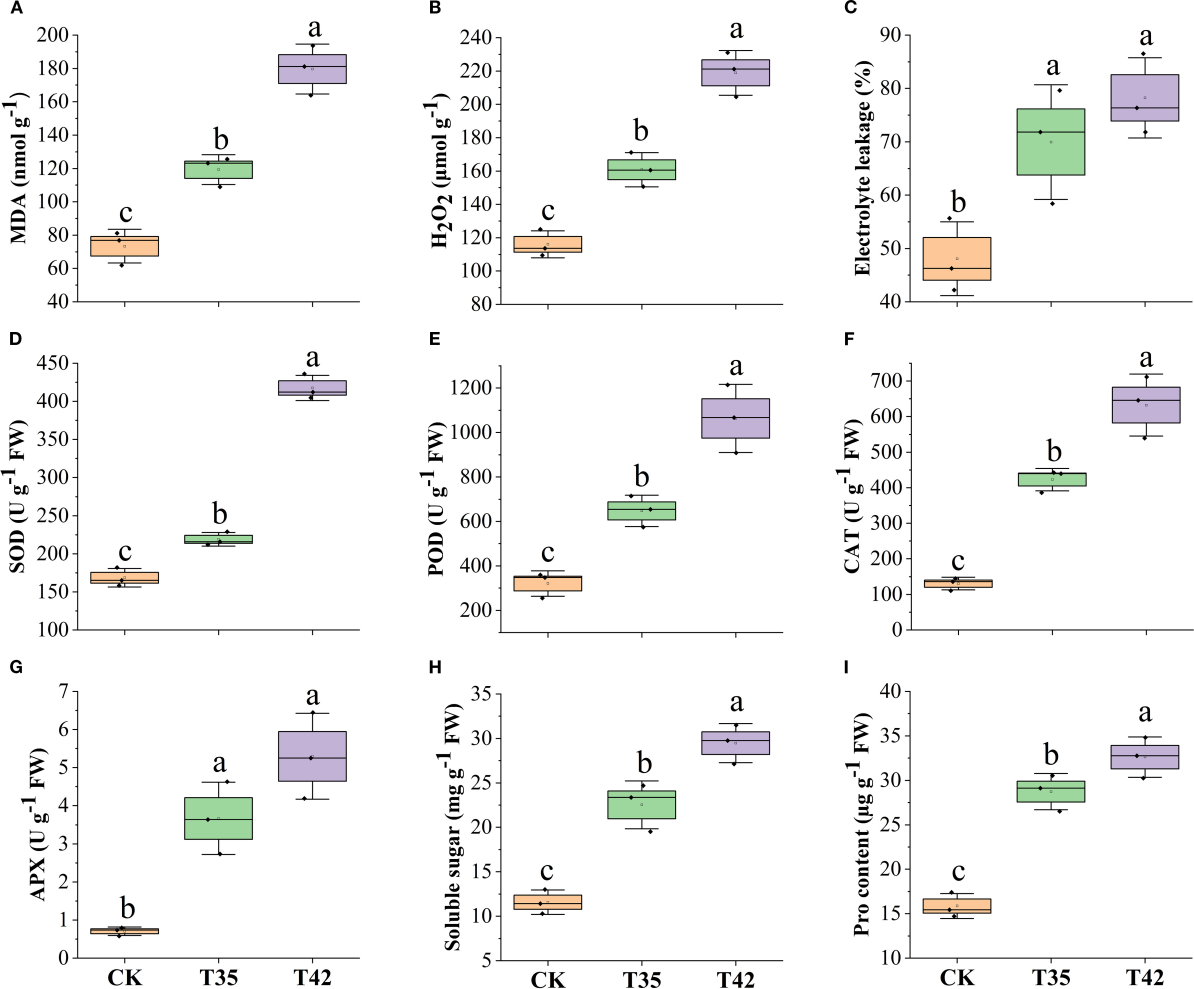
Estimation of high-temperature stress effect on ROS, antioxidants, and osmolytes activities of *R. moulmainense* **(A–F)**. The figure represents the effects of different treatments (CK, T35: 35°C, and T42: 42°C) on **(A–C)** ROS concentrations, **(D–F)** antioxidant activities, and osmolytes **(G–I)** under different treatments. Error bars show the standard error for each treatment across three replicates, while various alphabetic letters illustrate the significant differences as determined by the LSD test (*p <* 0.05).

High concentrations of ROS can impair chloroplast activity since they are mainly produced within the chloroplast. In order to better understand how *R. moumainense* reacts to heat stress, we examined ROS concentrations and antioxidant enzyme activity. In contrast to CK, plants under heat stress produced and accumulated high ROS, and the amounts of electrolyte leakage, H_2_O_2_, and MDA were remarkably elevated (**Figures 4A–C**). Antioxidant enzymes play a prominent role under any external stress in improving the thermotolerance of plants in combating ROS. After seven days, the heat stress at T42 significantly increased the levels of electrolyte leakage, H_2_O_2_, and MDA by 62%, 88%, and 144%, and interestingly, the concentrations of SOD, POD, and CAT were also elevated by 147%, 231%, and 384% in comparison to CK treatment, indicating that antioxidants tend to boost plants’ ability to withstand heat stress (**Figures 4D–F**). In addition, to counteract the adverse effects of ROS, the concentrations of osmolytes such as APX, soluble sugar, and proline were also increased by 651%, 154%, and 105% at T42 compared to CK treatment (**Figures 4G–I**).

### 3.4 RNA-Seq and DEGs Identification of transcriptome

**Figure 5:**
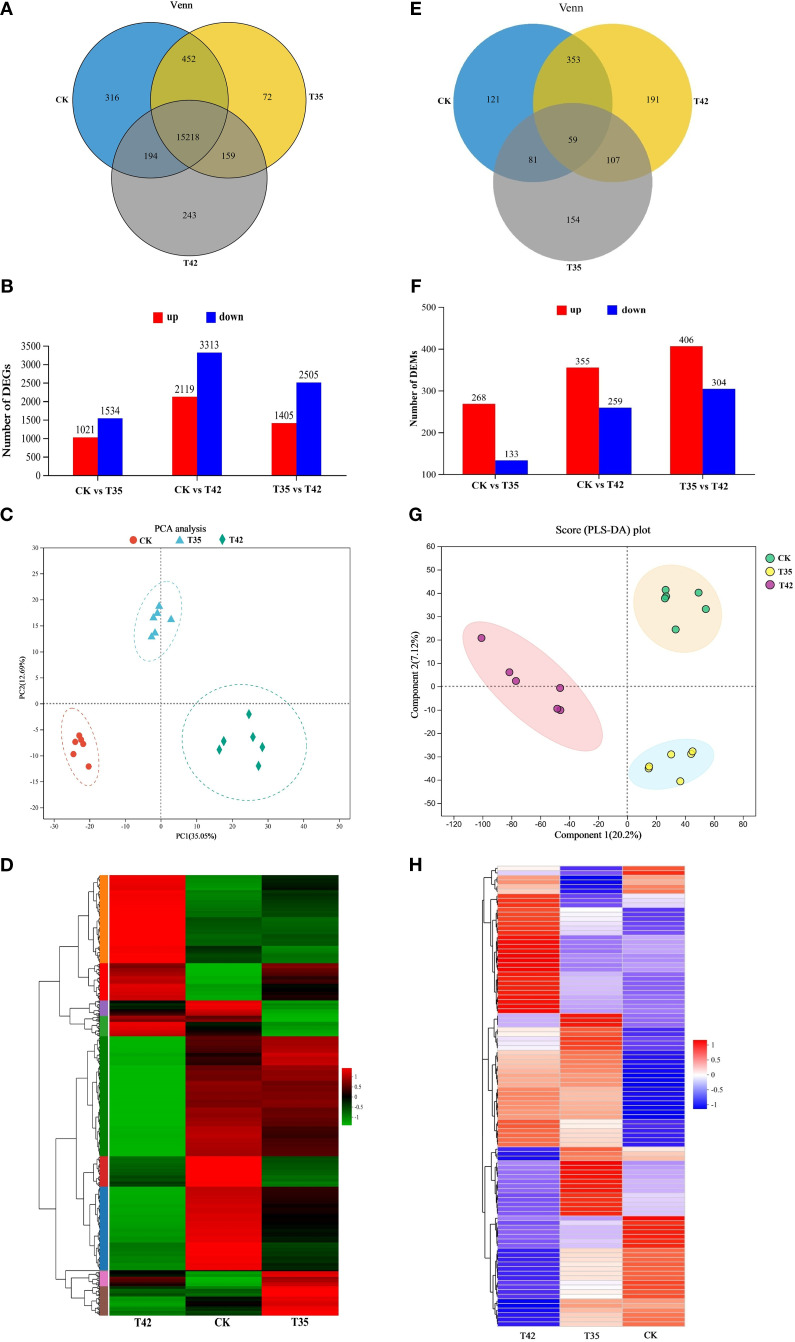
Illustration of transcriptome and metabolome analysis of *R. moulmainense* induced by high-temperature stress. **(A–H)** The figure A-D represents the effects of different treatments (CK, T35: 35°C, and T42: 42°C) on transcriptome analysis **(A)** Venn diagram analysis of DEGs, **(B)** the total number of upregulated and downregulated DEGs, the red color indicates upregulated DEGs, while the blue color indicates downregulated DEGs, **(C)** represent the PCA analysis of DEGs in each group, and **(D)** heat map of DEGs expressed in various groups. The figure **(E–H)** represents the effects of different treatments (CK, T35: 35°C, and T42: 42°C) on metabolome analysis **(E)** Venn diagram analysis of differentially accumulated metabolites (DAMs), **(F)** the total number of upregulated and downregulated DEMs, the red color indicates upregulated DAMs, while the blue color indicates downregulated DAMs, **(G)** represent the PLS-DA analysis of DAMs in each group, and **(H)** heat map of DAMs in various groups. The comparison groups were CK vs T35, CK vs T42, and T35 vs T42.

Overall, 45.07 million and 6806.44 million raw reads and bases were discovered, of which 6716.92 million were clean bases and 44.71 million were clean reads (**Supplementary Table S1**). Using a Venn diagram, we further evaluated the differential expression of genes in the CK, T35, and T42 groups to determine the total number of DEGs expressed. After seven days of heat stress, CK, T35, and T42 showed significant expression of 15218 DEGs, whereas CK, T35, and T42 showed significant expression of 316, 72, and 243 DEGs, respectively (**Figure 5A**). A pairwise comparison was employed to assess the up- and down-regulation of DEGs in order to analyze the corresponding treatments (**Figure 5B**). The red color indicates the upregulation of DEGs, whereas blue indicates the downregulation. The results of the DEGs were analyzed using principal component analysis (PCA), and all three treatments showed significant differences, explaining a total of 47.74% variation (**Figure 5C**). Furthermore, PC1 explained 35.05% of variation, while PC2 explained 12.69% among different treatments. The heat stress-induced DEGs were effectively annotated in the KEGG database under three distinct treatments, as evident in the heatmap analysis (**Figure 5D**). These results showed that the number of expressed DEGs was higher in T42 than in T35 compared to CK, suggesting that heat stress was more severe and effective at 42°C to cause such significant changes.

### 3.5 Metabolomic responses of *R. moulmainense* under high temperature stress

The metabolites of *R. moulmainense* were analyzed using LC-MS/MS to investigate the change in metabolomic profile in response to high temperature stress. Prior to data filtering, there were 1586 positive ions and 1269 negative ions in the raw data; following signal-to-noise ratio filtering, there were 1560 positive ions and 1207 negative ions (**Supplementary Table S2**). The screening criteria for differentiated metabolites were P<0.05 and VIP>1, and the screening conditions were fold change value (FC) (FC<1 or FC>1), using univariate statistical analysis (t-test) in conjunction with multivariate statistical analysis (OPLS-DA/PLS-DA) and recorded a total number of 3351 in T35 vs CK, 6119 in T42 vs CK, and 6332 differential metabolites in T35 vs T42 group (**Supplementary Table S3**). A Venn diagram was used to further assess the metabolite differential expression in the CK, T35, and T42 groups. A total of 59 DAMs were found in the mutual group, while 121, 154, and 191 DAMs were significantly expressed in the CK, T35, and T42 groups, respectively (**Figure 5E**). Comparing T35 and T42 vs the control group, the number of metabolites in T35 vs T24 group was higher, accounting for 406 upregulated and 304 downregulated metabolites (**Figure 5F**). Additionally, PLS-DA component 1 accounted for 20.2% of the variation, while component 2 explained 7.12%, demonstrating the clear and noteworthy differences between the various treatments (**Figure 5G**). The heatmap for the three different treatments shows that the heat stress-induced DAMs were successfully annotated in the KEGG database (**Figure 5H**).

### 3.6 GO and KEGG enrichment analysis of the DEGs and DAMs

**Figure 6:**
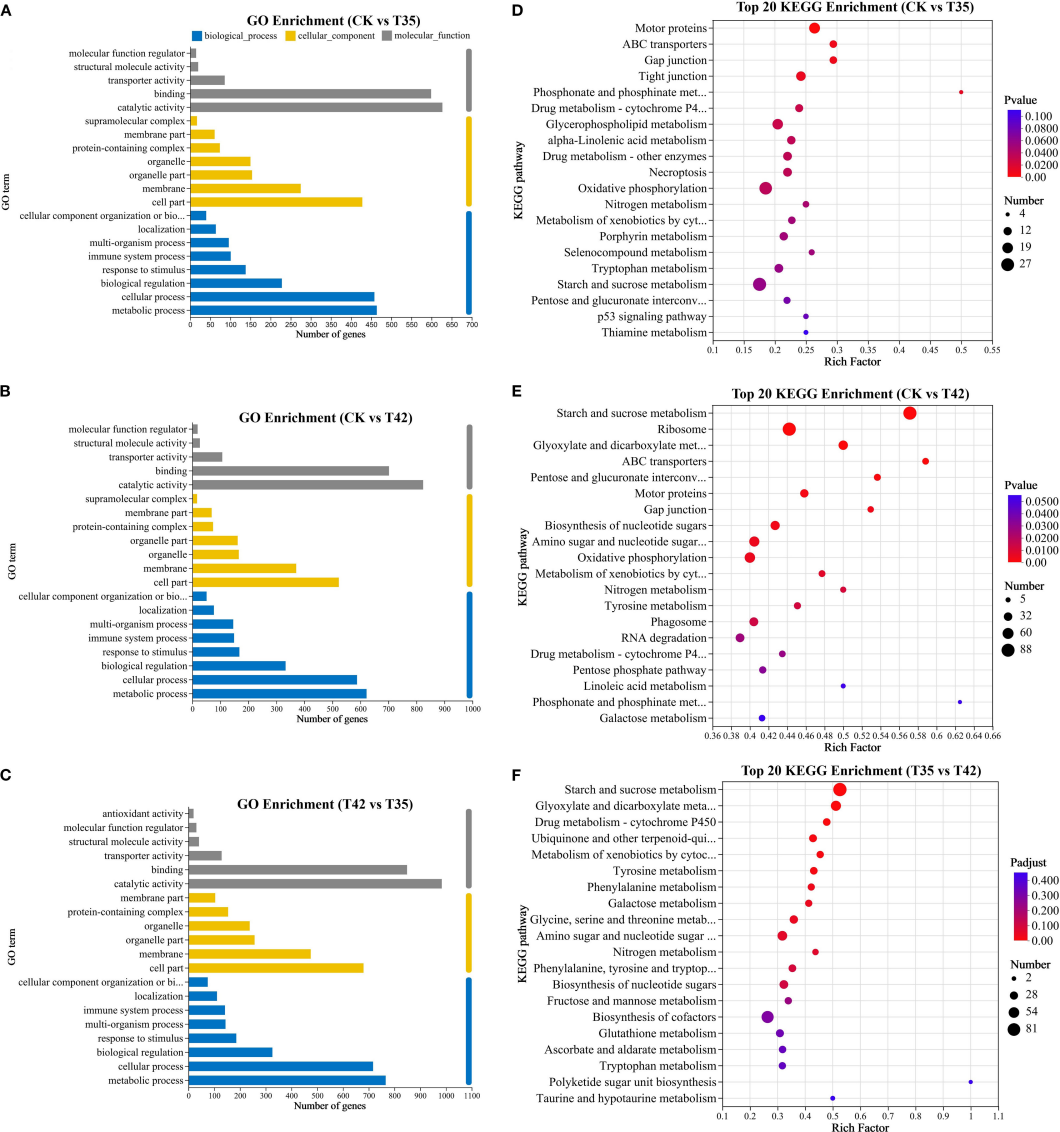
Illustration of transcriptome GO enrichment and KEGG enrichment analysis of *R. moulmainense* affected by high-temperature stress. **(A–F)** The figure represents the effects of different treatments (CK, T35: 35°C, and T42: 42°C) on **(A–C)** GO enrichment analysis and **(D–F)** KEGG enrichment analysis of top 20 enriched pathways, the axis represents the KEGG pathways, the abscissa displays the ratio of the number of DEGs annotated to the KEGG pathway to the total number of DEGs, and the size of the dots reflects the number of DEGs annotated to the KEGG pathway. The comparison groups were CK vs T35, CK vs T42, and T35 vs T42.

*R. moulmainense* plants treated for seven days under heat stress were subjected to Gene Ontology (GO) enrichment analysis for the CK group with T35 and T42. The GO enrichment analysis was used to categorize the DEGs into biological processes, cellular components, and molecular functions. The most enriched GO terms for biological processes in the CK vs T35 group were metabolic process, cellular process, and biological regulation (**Figures 6A–C**). The top three GO enrichments in terms of biological components were organelle, membrane, and cell part. The top three enriched GO keywords for molecular function were catalytic, binding, and transporter activity. The top three enrichments in biological process, cellular component, and molecular function in CK vs. T35 and T35 vs T42 groups were identical to those in the CK vs. T42 group (**Figures 6A–C**). According to KEGG analysis, DEGs were significantly linked to numerous metabolic pathways, with the metabolism of starch and sucrose, motor proteins, and ribosomes ranking as the top three enriched metabolic pathways (**Figures 6D–F**). The metabolism of starch and sucrose had the greatest number of genes in the CK vs. T42 and T35 vs. T42 comparison groups, but the CK vs. T35 group had the largest number of genes in the endoplasmic reticulum protein processing. The three groups that were compared mutually shared starch and sucrose metabolism and nitrogen metabolism pathways, while the ABC transporters pathway was found in the CK vs. T35 and T35 vs. T42 groups only (**Figures 6D–F**).

The DAMs were categorized using the GO enrichment analysis into three groups: metabolism, environmental information processing, and genetic information processing. These three metabolic enrichments—lipid metabolism, amino acid metabolism, and production of additional secondary metabolites—were identical in the CK vs. T35, T35 vs. T42, and CK vs. T42 groups (**Supplementary Figures S1A–C**). Similar to the DEGs identified in KEGG enrichments across the three groups, a considerable number of DAMs were also found for starch-sucrose metabolism and ABC transporters in the CK, T35, and T42 treatments (**Supplementary Figures S1D–F**).

### 3.7 Heat stress-induced starch-sucrose metabolism pathway

**Figure 7:**
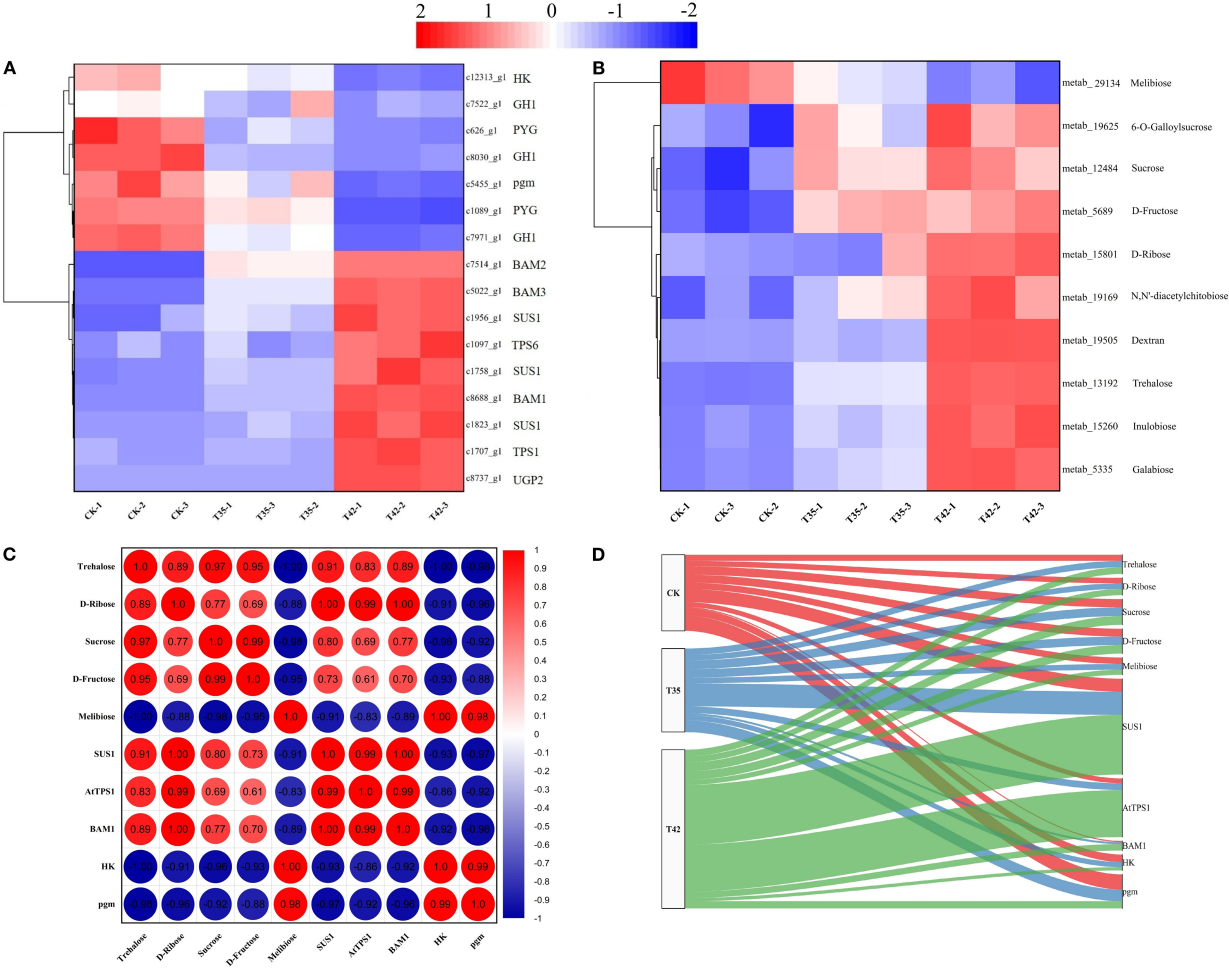
Analysis and correlation of DEGs and DAMs of *R. moulmainense* involved in starch-sucrose metabolism under high-temperature stress. The figure represents the effects of different treatments (CK; T35, 35°C, and T42, 42°C) on the DEGs **(A)** and DAMs **(B)** of the starch-sucrose metabolism pathway, as well as the correlation analysis between DEGs and DAMs **(C, D)** of *R. moulmainense* under high-temperature stress. The red color indicates high expression, while the blue color indicates low expression at *p* < 0.05; **p < 0.01; ***p < 0.001 **(A–C)**, and alluvial diagram **(D)**.

The heat stress-induced DEGs and DAMs of starch-sucrose metabolism were effectively annotated in the KEGG database under three distinct treatments (**Figures 7A, B**). Thus, DEGs and DAMs implicated in starch and sucrose metabolism were further examined. According to KEGG enrichment analysis, a total of sixteen DEGs were involved in the starch and sucrose metabolism pathway (**Figure 7A**). In contrast to stress treatments of T35 and T42, eleven genes were downregulated, and six were upregulated in the control treatment (**Figure 7A**). The number of genes upregulated in T35 was less than that in T42, and a total of nine genes were upregulated in T42 treatment (**Figure 7A**). The KEGG database significantly annotated the differentially accumulating primary metabolites caused by heat stress under three separate treatments that could trigger the secondary metabolites, and identified 10 metabolites (9 up-regulated and one down-regulated) in starch-sucrose metabolism (**Figure 7B**). The heatmaps of the DEGs and DAMs showed that the majority of the starch-sucrose metabolism genes and metabolites were elevated in T35 and T42, which could be the cause of the greater number of upregulated genes and metabolites in the moderate and high temperature stress treatments as compared to the control (**Figures 7A, B**). The DEGs and DAMs of starch-sucrose metabolism directly linked to heat stress were c1758_g1 (*SUS1*), c1707_g1 (*TPS1*), c8688_g1 (*BAM1*), metab_14581 (Trehalose), metab_15801 (D-ribose), metab_12484 (sucrose), and metab_5689 (D-fructose) exhibited the highest levels of expression, whereas c12313_g1(*HK*), c5455_g1(*pgm*), and metab_1630 (melibiose) were significantly downregulated under T42 (**Figures 7A, B**; **Supplementary Table S4**).

The correlation analysis for the combined transcriptome and metabolome was also performed further to evaluate the role of these significant DEGs and DAMs. The correlation analysis between DEGs and DAMs revealed that c1758_g1 (*SUS1*), c1707_g1 (*TPS1*), and c8688_g1 (*BAM1*), were positively correlated with metab_14581 (Trehalose), D-ribose (metab_15801), sucrose (metab_12484), and D-fructose (metab_5689) with a correlation coefficients higher than 0.6 and conversely c12313_g1(*HK*) and c5455_g1(*pgm*) were negatively correlated with them (**Figure 7C**). Alluvial analysis was used to visualize the correlations between DEGs and DAMs of starch-sucrose metabolism across different treatments (**Figure 7D**). The shifting of DEGs and DAMs throughout the three treatments is depicted in the alluvial diagram, and a significant proportion of DEGs and DAMs were transited to T42 in contrast to T35 and CK (**Figure 7D**). The alluvial diagram clearly shows a tendency of DEGs and DAMs shifting away from CK and toward T42, suggesting their prominent functions in the starch-sucrose metabolism pathway under high temperature-stress scenarios.

### 3.8 Heat stress triggered the ABC transporters metabolism pathway

**Figure 8:**
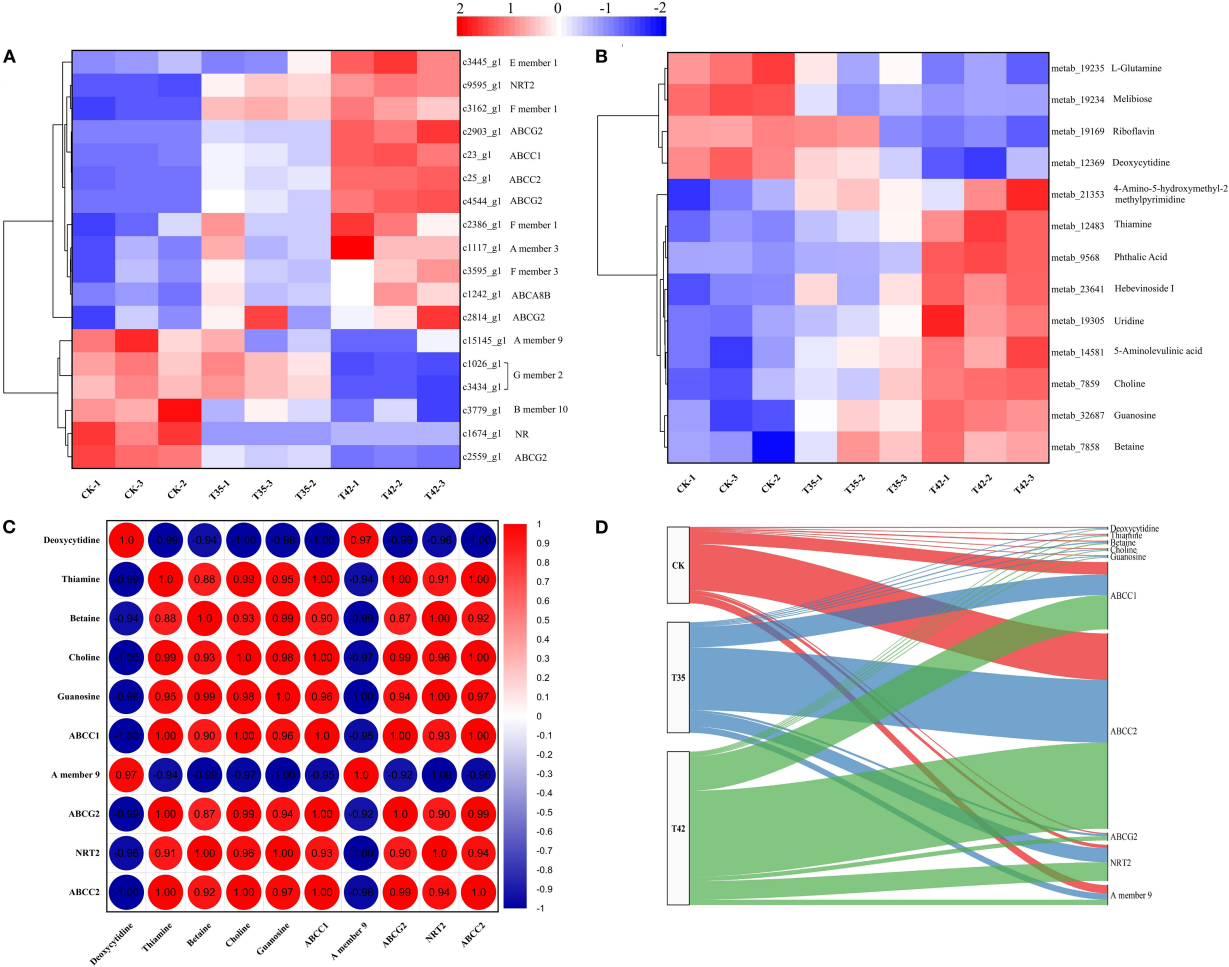
Analysis and correlation of DEGs and DAMs of *R. moulmainense* involved in ABC transporters metabolism under high-temperature stress. The figure represents the effects of different treatments (CK, T35: 35°C, and T42: 42°C) on the DEGs **(A)** and DAMs **(B)** of the ABC transporters metabolism pathway, as well as the correlation analysis between DEGs and DAMs **(C, D)** of *R. moulmainense* under high-temperature stress. The red color indicates high expression, while the blue color indicates low expression at *p* < 0.05; **p < 0.01; ***p < 0.001 **(A–C)**, and alluvial diagram **(D)**.

According to KEGG enrichment analysis, a total of 16 DEGs were associated with the ABC transporter, and 2 DEGs were involved in nitrogen metabolism across the three treatment groups in our study (**Figure 8A**). Five DEGs related to ABC transporters were elevated, and eleven DEGs were downregulated in the control group (**Figure 8A**). Furthermore, four DEGs involved in ABC transporters were upregulated, while twelve were downregulated in the T35 treatment (**Figure 8A**). In comparison to the control and T35, eleven DEGs of ABC transporters were upregulated, and five were downregulated in T42, indicating that these genes may play a vital role during heat stress. Regarding nitrogen metabolism, DEG encoding *NRT2* was also upregulated in T42 to modulate the metabolic pathway of the ABC transporters (**Figure 8A**). The KEGG database significantly annotated the differentially accumulating primary metabolites that could regulate secondary metabolites caused by heat stress under three separate treatments, and 13 (9 up-regulated and 4 down-regulated) were found in ABC transporter metabolism under the T42 high-temperature stress treatment compared to the control treatment (**Figure 8B**). Compared to T45, T35 showed fewer DAMs that were elevated, indicating that these pathways were activated more strongly during high temperature stress than during moderate stress (**Figure 8B**). The highest levels of expression in the DEGs and DAMs of ABC transporters metabolism were found in c23_g1 (*ABCC1*), c25_g1 (*ABCC2*), c2903_g1 (*ABCG2*), c9595_g1 (*NRT2*), metab_12483 (Thiamine), metab_7858 (Betaine), metab_7859 (Choline), and metab_32687 (Guanosine) (**Figures 8A, B**). In contrast, c15145_g1 (*A member 9*) and metab_12369 (Deoxycytidine) were significantly downregulated under T42 compared to the control treatment, demonstrating a direct link between heat stress regulation and the response of *R. moulmainense* (**Figures 8A, B**; **Supplementary Table S4**).

To further assess the role of these essential DEGs and DAMs, a correlation analysis was also carried out for the combined transcriptome and metabolome (**Figures 8C, D**). In comparison to CK, the correlation analysis between DEGs and DEMs of ABC transporters metabolism pathway, c23_g1 (ABCC1), c25_g1 (ABCC2), c2903_g1 (ABCG2), and c9595_g1 (*NRT2*) had positive correlations with metab_12483 (Thiamine), metab_7858 (Betaine), metab_7859 (Choline), and metab_32687 (Guanosine) with correlation coefficients greater than 0.9, while c15145_g1 (A member 9) had a negative correlation with them under T42 treatment (**Figure 8C**). In contrast to T35 and CK, a considerable proportions of DEGs and DAMs were transited to T42, as shown by alluvial analysis, which further demonstrates the correlations between DEGs and DAMs of ABC transporters metabolism (**Figure 8D**). According to the alluvial analysis, DEGs and DAMs have a tendency to move toward T42 as compared to CK, indicating that they play essential roles in the ABC transporter metabolic pathway under conditions of high temperature stress (**Figure 8D**).

## 4 Discussion

*R. moulmainense*, an evergreen alpine azalea, grows best in chilly, humid woodland with acidic soil, representing a temperate climate. As a result, during domestication, high-altitude rhododendrons struggle to adapt to the hot, dry metropolitan summer climate. Consequently, it is difficult for high-altitude rhododendrons to acclimate to the hot, dry urban summer climate. Temperature is a major factor in low-altitude settings; however, the possible mechanisms of *R. moulmainense* reacting to stress caused by high temperatures remain unclear. Thus, we conducted an integrated analysis of the transcriptome, biochemical, and physiological characteristics of *R. moulmainense* under high-temperature stress. Our research provides a strong basis for identifying the growth-regulating pathways under high-temperature stress that could enable the successful cultivation and acclimatization of high-altitude rhododendrons in low-altitude areas.

Plant growth status under inevitable external stress reflects the accumulation of adenosine triphosphates (ATPs) from the interaction of several different processes, including photosynthesis, transpiration, and carbon metabolism. Plants adjust their chlorophyll synthesis, photosynthetic efficiency, and carbon metabolism to sustain a constant carbon supply and ensure plant growth under stressful conditions (Flexas et al., 2004). Thus, the current study hypothesized how different metabolic pathways interact to induce *R. moulmainense’s* starch and sucrose metabolism under high-temperature stress. Under high-temperature stress, *R. moulaminense* leaves had reduced concentrations of chlorophyll fluorescence, chlorophyll contents and chlorophyll pigment, and photosynthetic rates, with the exception of CO_2_ ci, which was higher (**Figures 1A–L**). High temperatures can harm the photosynthetic apparatus and diminish the efficiency of light energy conversion by denaturing the proteins that regulate PSII and lowering the maximum quantum yield of PSII (Fv/Fm) (Kim et al., 2015; Tian et al., 2017). Chlorophyllase and other vital enzymes in the chlorophyll production pathway are heat-sensitive, which causes chlorosis (leaf yellowing) and decreased photosynthetic capacity (Hörtensteiner, 2006). A previous study reported that a higher CO_2_ concentration and reduced plant absorption of light were responsible for the decreased chlorophyll concentrations in wheat (Zhang et al., 2022). The higher concentration of CO_2_ ci (**Figure 1L**) suggests the increased accumulation of CO_2_ during respiration, while at high temperatures, the stomata tend to close and release less CO_2_, increasing the CO_2_ ci rate.

Various environmental factors can induce the leaf’s anatomical, stomata, and cell ultrastructural characteristics (**Figures 2**, **3**), which are associated with variations in leaf gas exchange and can hinder the transport of CO_2_ and water, affecting photosynthetic traits (Khan et al., 2021). The chloroplast, which is the photosynthetic apparatus, contains chlorophyll; however, the high-temperature stress negatively affected the chloroplast and cell structure, which may have decreased the amount of chlorophyll and slowed down the rate of photosynthesis. Drought and high temperatures frequently occur together, which declines stomatal conductance and increases membrane fluidity, upsetting the structure of photosynthetic complexes and decreasing their efficiency (Baker, 2008; Hörtensteiner, 2006). High temperatures cause peroxidation and increase the formation of reactive oxygen species (ROS), resulting in the leakage of cellular contents and further decreasing the amounts of proteins, lipids, and chlorophyll, which lowers photosynthetic efficiency (Mittler, 2002). This study found that high-temperature stress oxidized cell membrane lipids to limit normal cell division (**Figures 3G–I**), decreased the efficiency of leaf photosynthetic processes (**Figures 1I–L**), and increased the activities of antioxidant enzymes and osmolytes (**Figures 4D–I**) to scavenge ROS (**Figures 4A–C**) in *R. moulmainense* plants. In addition to producing oxidative damage, an excess ROS also boosts antioxidant system activity by encouraging the synthesis of proteins and enzymes linked to stress adaptation (Choudhury et al., 2017). Plants produce a variety of antioxidant enzymes and osmolytes, and the current study found that their higher activities were directly proportional to the ROS concentrations (**Figures 4A–F**). Our results are in line with Liu et al. (2024), who demonstrated that high-temperature stress increased the ROS and antioxidant activities in *R. moulmainense*.

Glucose, as Starch-derived soluble sugars regulating plant secondary metabolism, is thought to be the primary byproducts of photosynthesis that control plant growth and development and functions as an osmoprotectant, protecting proteins and membranes under high-temperature stress (Chen et al., 2023; Baker, 2008). The current results of elevated activities of Rubisco, CS, SS, and SPS (**Figures 1M–P**) under high-temperature stress might be correlated with the fact that, upon exposure to high temperatures, plants modify the carbon partitioning between sucrose and starch to maximize the use of available resources. Under high-temperature stress, sucrose functions as a signaling molecule that builds up in cells and alters the expression of genes responsive to stress, including heat shock proteins (HSPs), which support plant growth and repair metabolic processes by regulating cell turgor and protecting cellular structures (Baniwal et al., 2004; Ruan, 2014). RuBisCO accounts for 20–50% of the total soluble protein in plant leaves and contributes significantly to the growth and development under stress conditions (Parry et al., 2003). In order to ensure that energy is allocated to support growth and repair activities under stress, high temperatures frequently result in increased sucrose production and export to sink tissues (Ruan, 2014). The current findings of increased concentrations of sugar enzymes in stressed plants may suggest the regulation mechanism of starch and sucrose metabolism to enhance the growth performance of *R. moullmainense* under high-temperature stress.

**Figure 9:**
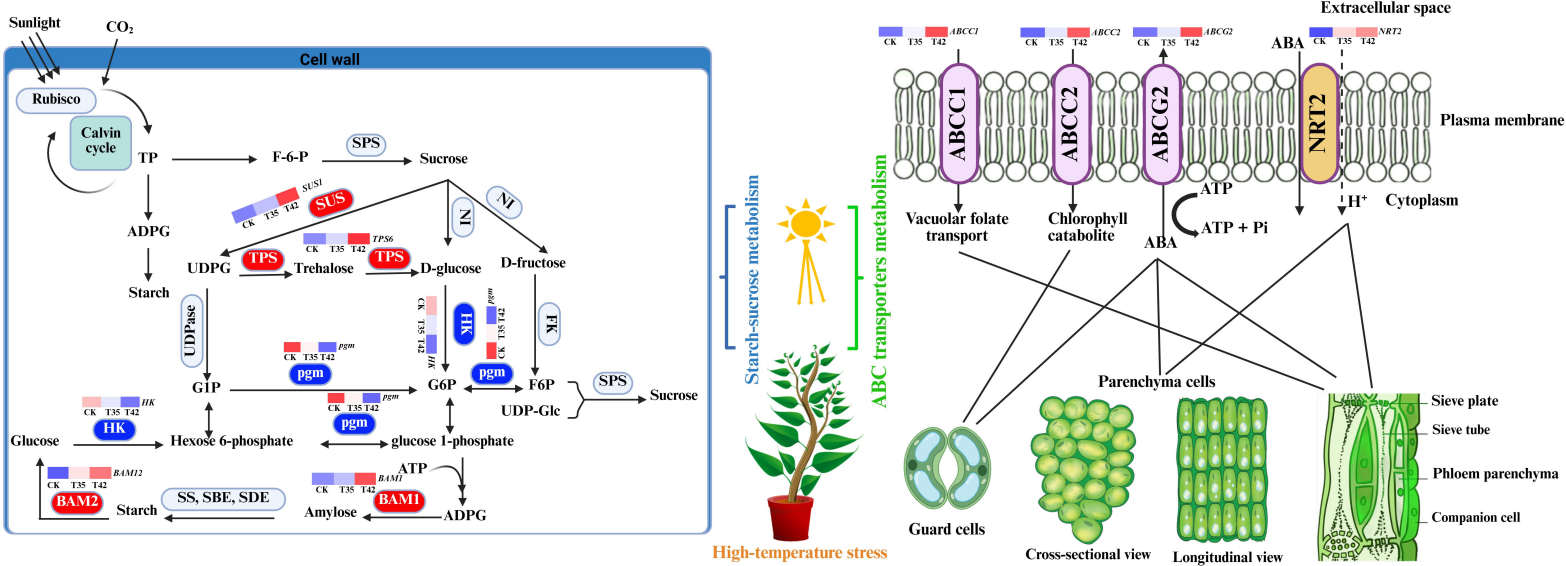
Illustration of metabolic network of starch-sucrose metabolism and ABC transporters metabolism pathways. ADPG, ADP-glucose; G1P, Glc-1-phosphate; G6P, Glc-6-phosphate; F6P, fructose-6-phosphate; UDP-Glc, uridine diphosphate glucose; SPS, Sucrose phosphate synthase; NI, neutral invertase; SUS, sucrose synthase; HK, hexokinase; FK, fructokinase; pgm, phosphoglucomutase; UDPase, UDP-glucose pyrophosphorylase; SS, starch synthase; SBE, starch branching enzyme; SDE, starch debranching enzyme; BAM, beta-amylase. The encoding DEGs for the enzymes in the red rectangles were upregulated, and downregulated for enzymes in the blue rectangles.

In the current study, we discovered DEGs and DAMs for the starch-sucrose metabolism and ABC transporters pathways, demonstrating their critical functions in plant secondary metabolism under high-temperature stress (**Figures 7A, B**). In the transformation of starch and sucrose, the DEGs and DAMs of UTP-glucose-1-phosphate uridylyltransferase (UGP2), sucrose synthase (SUS1), trehalose 6-phosphate (AtTPS1), and beta-amylase (BAM1) were upregulated, while the genes beta-glucosidase (GH1), hexokinase (HK), phosphoglucomutase (pgm), and glycogen phosphorylase (PYG) were downregulated (**Figures 8A, B**). The KEGG database extensively identified the differentially expressed genes and metabolites involved in starch-sucrose metabolism, demonstrating the potential network of these pathways in influencing the growth of *R. moulmainense* under high-temperature stress (**Figure 9**). This suggests that the starch and sucrose metabolism pathway may significantly regulate the growth of *R. moulmainense* under high-temperature stress (**Figure 9**). According to a previous study, BAM1 facilitates the breakdown of starch to release carbon skeletons for proline and soluble sugars synthesis, which are critical for osmotic adjustment and oxidative defense under heat stress (Zanella et al., 2016). Our findings are consistent with (Ponnu et al., 2011), who demonstrated that T6P may regulate plant growth and development through a mechanism involving the coordination of ABA and sugar metabolism. A crucial enzyme for sugar metabolism, SUS regulates carbon partitioning in plants and influences significant agronomic characteristics as well as abiotic reactions to stress (Xiao et al., 2024). Sugar metabolism is a complex network of pathways that performs various functions in plants (Halford et al., 2011). Sugar buildup in plants depends on two mechanisms: biosynthesis and transport. Some studies have suggested that genes involved in sugar transport are crucial for sugar accumulation, while other investigations have concluded that genes involved in sugar biosynthesis are essential for sugar accumulation (Wang et al., 2016). Thus, to enhance the plant’s growth regulatory mechanism through sugar accumulation, we also investigated the interlinked pathways of ABC transporters and nitrogen metabolism (**Figures 8A**, **9**). Starch and sucrose metabolism produce ATP and NADPH, which are essential for the function of ABC transporters and nitrogen utilization (Zeeman et al., 2010).

ABC transporters help in the supply of ABA and antioxidants, a crucial hormone for stress signaling and stomatal control, and enzymes that scavenge ROS under the conditions of high temperature (Kuromori et al., 2010; Wang et al., 2016). The DEGs and DAMs for ABC transporters ABCC1, ABCC2, and ABCG2 were identified and upregulated in the current study, indicating their crucial role in the ABA efflux and influx in controlling high-temperature stress (**Figures 8A, B**, **9**). The results showed that DEGs of ABC transporters and nitrogen metabolism play a crucial role in modulating ABA to regulate plant growth under high-temperature stress (**Figure 9**). When plants are subjected to water deficiency conditions, ABA synthesis increases quickly in the vascular parenchyma cells of roots and shoots, despite the accumulation being restricted in guard cells (Endo et al., 2008). Different transporters, including ABA transporters, participate in the process of regulating guard cell movement, which improves heat tolerance in plants by reducing water loss (Jarzyniak and Jasiński, 2014). In order to reduce oxidative stress induced by heat, ABC transporters facilitate the supply of secondary metabolites such as flavonoids and antioxidants (Petrussa et al., 2013). ABC transporters facilitate nitrogen absorption, guaranteeing a consistent flow of nitrogen-containing building blocks for chlorophyll synthesis and, ultimately, photosynthesis (Tegeder and Rentsch, 2010). It is suggested that the ABA supply is not unidirectional (Parry et al., 2003; Smeekens et al., 2010), as it is a sesquiterpene-structured isoprenoid plant hormone that is generated at numerous locations and delivered to the site of action by ABCG transporters (Campbell et al., 2003). In this study, we also identified the upregulation of *NRT2* gene, which might correlate with their regulation mechanism of ABA to modulate plant growth (**Figure 8A**). NRT2 transporters support nitrate absorption and stomatal activity by adjusting ABA levels, which control the plant’s response to heat stress (Wilkinson and Davies, 2002).

Analyses of the transcriptome and metabolome were correlated to gain a more thorough understanding of the metabolic processes and gene expression resulting plant secondary metabolism of *R. moulmainense* under high-temperature stress (**Figures 7C, D**, **8C, D**). According to the KEGG pathway enrichment analysis, the starch-sucrose metabolism and ABC transporters pathways were considerably enriched under high-temperature stress, and several DEGs and DAMs also showed positive correlations with these pathways, whereas a small number showed negative correlations (**Figures 7**, **8**). The KEGG findings annotated a significant number of DEGs and DAMs, indicating their possible role and functions in the starch-sucrose metabolism pathway (**Figure 9**). Neutral invertase (NI) transforms sucrose into fructose and glucose, which are then further transformed by fructokinase (FK) and hexokinase (HK) into fructose-6-phosphate (F6P) and Glc-6-phosphate (G6P), respectively (**Figure 9**). Furthermore, sucrose synthase (SUSY), UDP-glucose pyrophosphorylase (UDPase), and phosphoglucomutase (PGM) catalyze three consecutive processes that can also convert sucrose to G6P. Starch synthesis uses both G1P and G6P, while suc-phosphate synthase (SPS) and suc-phosphate phosphatase (SPP) can re-synthesize sucrose using F6P in combination with UDP-glucose (UDPG) (**Figure 9**). Furthermore, phosphoglucose isomerase (PGI) allows G6P and F6P to interconvert into one another. BAM (beta-amylase) and SS (starch synthase), SBE (starch branching enzyme), and SDE (starch debranching enzyme) convert ADPG (aDP-glucose) into starch, which is then converted into glucose via BAM (**Figure 9**). ATP-binding cassette (ABC) transporters positively interacted with other membrane-bound transporters to stimulate the movement of ABA from the extracellular space to the cytosol under high-temperature stress; *AABCC2* is an ABA inflow transporter; *ABCG2* is an ABA exporter; *ABCC1* is an ABA efflux transporter; and *NRT2* is a low-affinity nitrate transporter that can also regulates stomatal aperture through its ABA-importing function (**Figure 9**). These findings suggested that the DEGs and DAMs implicated in the ABC transporter and starch-sucrose metabolism pathways (**Figure 9**) could improve *R. moulmainense*’s ability to withstand high-temperature stress. A significant regulatory network of genes of the ABC transporters pathway has previously been revealed to contribute to the accumulation of carbohydrate metabolites that are involved in glucose metabolism and response to heat stress (Campbell et al., 2003; Manzi et al., 2015).

Although this study sheds light on insightful mechanisms underlying rhododendron heat tolerance, future research studies should take into account several limitations. First, the experiments were carried out in a controlled setting, which would not represent the complexity of real field settings where heat stress might also interact with other factors, such as drought, pathogens and fluctuating light levels. Second, the study mainly examined the findings based on a single type of cultivar, which could have limited the findings’ applicability to other temperate plants. Third, long-term observational effects like survival under extended heat stress were not assessed, despite evaluating the mechanisms of physiological and biochemical reactions in a short period. To determine the comprehensive understanding of heat tolerance processes, multi-stress and long-term studies are required to evaluate the integrated insights of the microbiome, metabolome, and transcriptome.

## 5 Conclusion

This study found that high-temperature stress adversely affects the plant’s anatomy, stomatal traits, cell ultrastructure, chlorophyll synthesis, and photosynthesis. The findings of photosynthetic traits, ROS, and antioxidants revealed significant correlations among physiological, biochemical, and molecular traits. Several genes and metabolites linked to starch-sucrose metabolism and ABC transporter metabolism pathways were discovered to be involved in the *R. moulmainense* response to high-temperature stress. The potential genes and metabolites of starch-sucrose metabolism and ABC transporters were identified in the interlinked pathways that can promote the growth-regulative mechanism of *R. moulmainense* under high-temperature stress. However, further studies are needed to elaborate on how these metabolic pathways can be regulated and improved to enhance the cultivation and domestication of Rhododendrons. These potential pathways can serve as future targets to uncover the responsible candidates and approaches for enhancing the regulatory pathways of *R. moulmainense* under high-temperature stress. Future studies could investigate agronomic techniques such as optimizing irrigation schedules, utilizing mulching and shading tactics, and introducing beneficial microbes like mycorrhizal fungi and breeding methods, such as selection of genotypes with improved water-use characteristics and thermal tolerance, as well as using the identified upregulated DEGs under heat stress as future candidates in the preparation of heat-tolerant Rhododendron species.

## Data Availability

The datasets presented in this study can be found in online repositories. The names of the repository/repositories and accession number(s) can be found in the article/**Supplementary Material**.
