# Supplementary material for: Growth regulation mechanism of Rhododendron moulmainense to high-temperature stress: integrated physiological, transcriptomic, and metabolomic insights
Source: Front Plant Sci. 2025 Oct 7;16:1680853. doi: 10.3389/fpls.2025.1680853 (PMC12538711; doi:10.3389/fpls.2025.1680853)
Supplement: Supplementary file 1 [file DataSheet1.docx]

| **Sample** | **Total reads** | **Total bases** | **Clean reads** | **Clean bases** | **Q20 rate(%)** | **Q30 rate(%)** | **GC(%)** |
| --- | --- | --- | --- | --- | --- | --- | --- |
| CK-1 | 41343124 | 6242811724 | 40984568 | 6164869560 | 98.6 | 95.8 | 46.74 |
| CK-2 | 47567406 | 7182678306 | 47184134 | 7094843167 | 98.65 | 95.92 | 46.62 |
| CK-3 | 41831434 | 6316546534 | 41520826 | 6251331428 | 98.59 | 95.71 | 46.43 |
| CK-4 | 43661268 | 6592851468 | 43320774 | 6514828018 | 98.63 | 95.86 | 46.7 |
| CK-5 | 45669206 | 6896050106 | 45290242 | 6812458273 | 98.65 | 95.92 | 46.34 |
| CK-6 | 43176574 | 6519662674 | 42847864 | 6448311001 | 98.65 | 95.86 | 46.98 |
| T35-1 | 45401888 | 6855685088 | 45045672 | 6777924231 | 98.63 | 95.84 | 47.04 |
| T35-2 | 43369580 | 6548806580 | 43030112 | 6476471337 | 98.61 | 95.78 | 46.93 |
| T35-3 | 42589764 | 6431054364 | 42271872 | 6355189854 | 98.68 | 96 | 47.34 |
| T35-4 | 40724012 | 6149325812 | 40392820 | 6070558226 | 98.61 | 95.81 | 47.18 |
| T35-5 | 42604560 | 6433288560 | 42260674 | 6346152671 | 98.66 | 95.98 | 47.58 |
| T35-6 | 46877262 | 7078466562 | 46523554 | 6976675021 | 98.67 | 95.99 | 47.55 |
| T42-1 | 43904710 | 6629611210 | 43569116 | 6537484110 | 98.7 | 96.06 | 47.51 |
| T42-2 | 41165884 | 6216048484 | 40849202 | 6145635046 | 98.6 | 95.78 | 47.02 |
| T42-3 | 50051080 | 7557713080 | 49630514 | 7442672173 | 98.8 | 96.37 | 47.49 |
| T42-4 | 49917854 | 7537595954 | 49505294 | 7414381457 | 98.81 | 96.41 | 47.54 |
| T42-5 | 48437210 | 7314018710 | 48034520 | 7200450774 | 98.75 | 96.23 | 47.59 |
| T42-6 | 52410002 | 7913910302 | 51954100 | 7773021849 | 98.78 | 96.31 | 47.69 |
| Total | 45039045.4 | 6800895862 | 44678658.78 | 6711292122 |  |  |  |
|  | 45.0390454 | 6800.895862 | 44.67865878 | 6711.292122 |  |  |  |

**Table S1. RNA-Seq total and clean reads data**

**Table S2. LC-MS/MS metabolome data**

| **Ionic mode** | **Effective Peak (RAW)** | **Metabolites identified (RAW)** | **Proportion (%)** | **Effective Peak (Origin)** | **Metabolites identified (origin)** | **Proportion (%)** |
| --- | --- | --- | --- | --- | --- | --- |
| Positive | 14947 | 1586 | 10.61% | 13655 | 1560 | 11.42% |
| Negative | 18170 | 1269 | 6.98% | 14601 | 1207 | 8.27% |

**Table S3.** [**Differential metabolite analysis information**](http://analysis.majorbio.com/metab/expdiff_overview/task_id/i60f_5f8kaq3diquvklrkr31av9) **data.**

| **Diff Group** | **Total Diff Number** | **Up** | | **Down** | |
| --- | --- | --- | --- | --- | --- |
| T35 vs CK | 3951 (401) | 2403 (268) | | 1548 (133) | |
| T42 vs CK | 6119 (614) | 3051 (355) | | 3068 (259) | |
| T35 vs T42f | 6332 (710) | | 3119 (406) | | 3213 (304) |


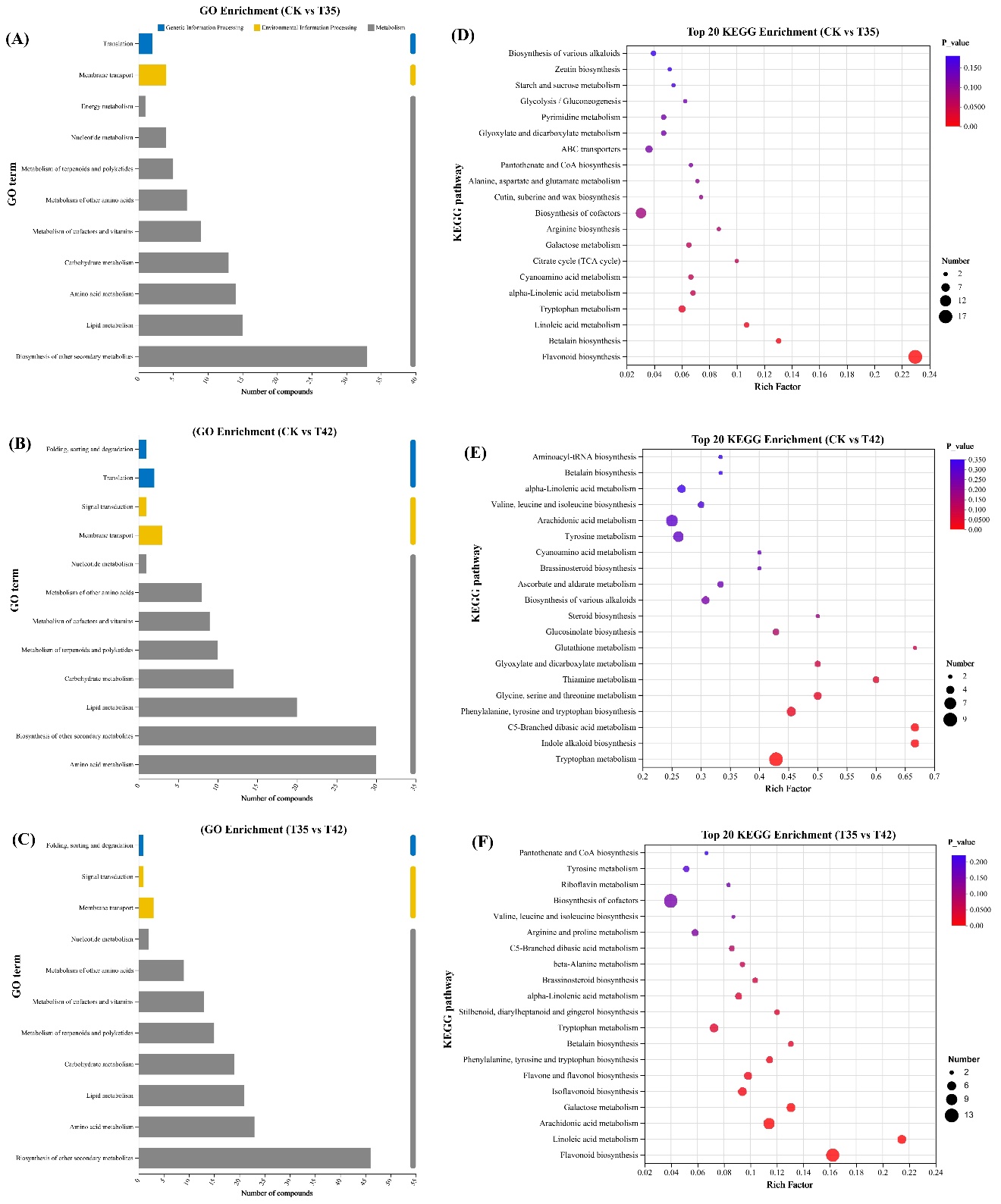


**Supplemental Figure S1.** Illustration of metabolome GO enrichment and KEGG enrichment analysis of *R. moulmainense* affected by high-temperature stress. (A-F) The figure represents the effects of different treatments (CK: 25ºC, T35: 35ºC, and T42: 42ºC) on (A-C) GO enrichment analysis and (D-F) KEGG enrichment analysis of top 20 enriched pathways, the axis represents the KEGG pathways, the abscissa displays the ratio of the number of DAMs annotated to the KEGG pathway to the total number of DEGs, and the size of the dots reflects the number of DAMs annotated to the KEGG pathway. The comparison groups were CK vs T35, CK vs T42, and T35 vs T42.


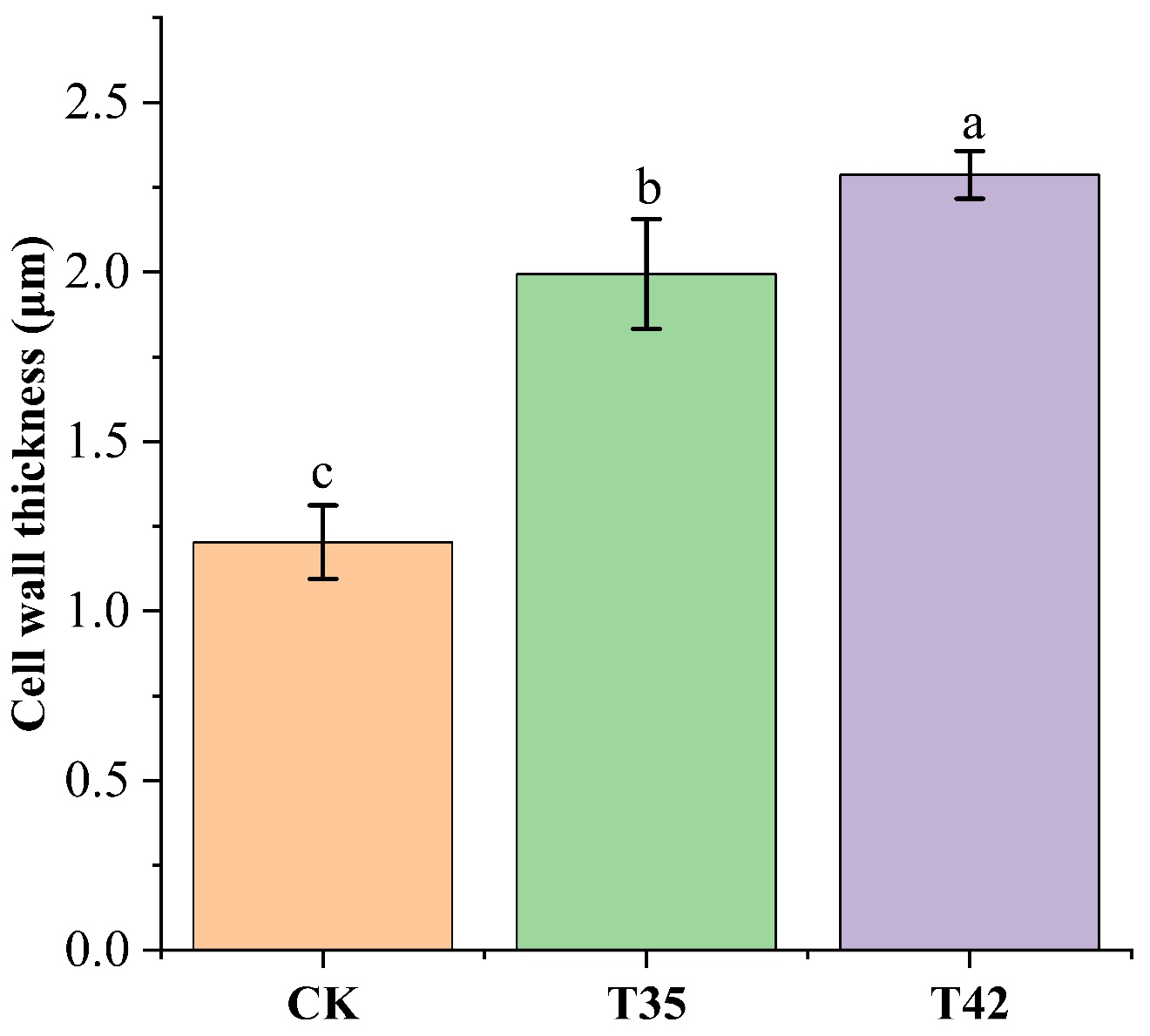


**Supplemental Figure S2.** Impact of high-temperature stress on leaf cell wall thickness of *R. moulmainense*. (A-C) Error bars show the standard error for each treatment across three replicates, while various alphabetic letters illustrate the significant differences as determined by the LSD test (*p <* 0.05). CK; T35: 35 ºC; and T42: 42 ºC.
