# Supplementary figures and images for: Growth regulation mechanism of Rhododendron moulmainense to high-temperature stress: integrated physiological, transcriptomic, and metabolomic insights
Source: Front Plant Sci. 2025 Oct 7;16:1680853. doi: 10.3389/fpls.2025.1680853 (PMC12538711; doi:10.3389/fpls.2025.1680853)

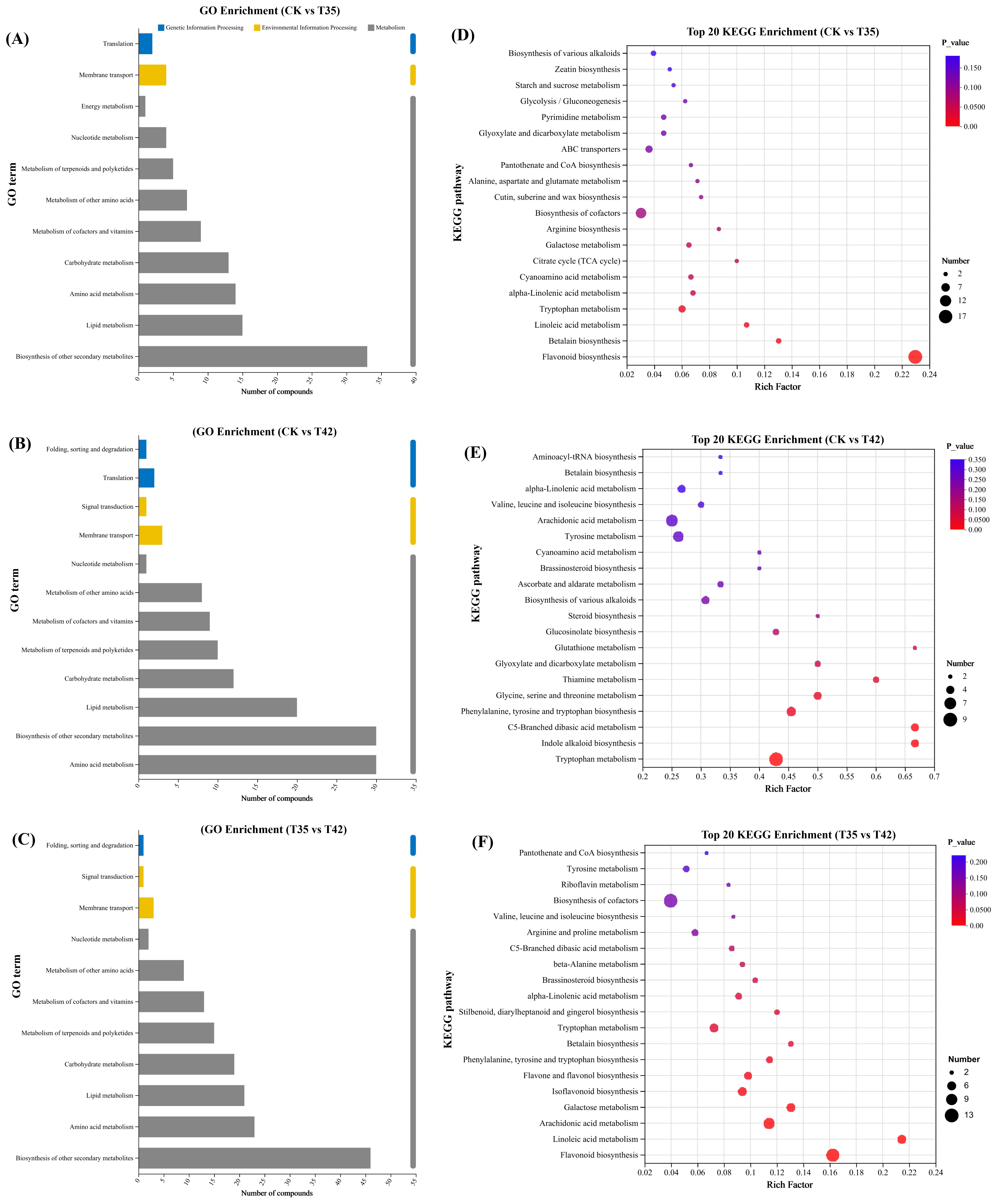

Supplement: Supplementary file 2 [file Image1.jpg]
